# Delivery of telehealth nutrition and physical activity interventions to adults living in rural areas: a scoping review

**DOI:** 10.1186/s12966-023-01505-2

**Published:** 2023-09-15

**Authors:** Jaimee Herbert, Tracy Schumacher, Leanne J. Brown, Erin D. Clarke, Clare E. Collins

**Affiliations:** 1 School of Health Sciences (Nutrition and Dietetics), Department of Rural Health, University of Newcastle, 114/148 Johnston St, North Tamworth, NSW 2340 Australia; 2 School of Health Sciences (Nutrition and Dietetics), University of Newcastle, ATC 205, ATC Building, University Drive Callaghan, Newcastle, NSW 2308 Australia; 3 School of Health Sciences (Nutrition and Dietetics), University of Newcastle, ATC 310, ATC Building, University Drive Callaghan, Newcastle, NSW 2308 Australia

**Keywords:** Nutrition, Physical activity, Telehealth, Rural

## Abstract

**Background:**

Lifestyle behaviours related to smoking, alcohol, nutrition, and physical activity are leading risk factors for the development of chronic disease. For people in rural areas, access to individualised lifestyle services targeting behaviour change may be improved by using telehealth. However, the scope of literature investigating telehealth lifestyle behaviour change interventions for rural populations is unknown, making it difficult to ascertain whether telehealth interventions require adaptation for rural context via a systematic review. This scoping review aimed to address this gap, by mapping existing literature describing telehealth lifestyle interventions delivered to rural populations to determine if there is scope for systematic review of intervention effectiveness in this research topic.

**Methods:**

The PRISMA extension for scoping review checklist guided the processes of this scoping review. A search of eight electronic databases reported in English language until June 2023 was conducted. Eligible studies included adults (18 years and over), who lived in rural areas of high-income countries and undertook at least one synchronous (video or phone consultation) telehealth intervention that addressed either addictive (smoking or alcohol), or non-addictive lifestyle behaviours (nutrition or physical activity). Studies targeting addictive and non-addictive behaviours were separated after full text screening to account for the involvement of addictive substances in smoking and alcohol studies that may impact behaviour change interventions described. Studies targeting nutrition and/or physical activity interventions are presented here.

**Results:**

The search strategy identified 17179 citations across eight databases, with 7440 unique citations once duplicates were removed. Full texts for 492 citations were retrieved and screened for inclusion with 85 publications reporting on 73 studies eligible for data extraction and analysis. Of this, addictive behaviours were comprised of 15 publications from 13 studies. Non-addictive behaviours included 70 publications from 58 studies and are reported here. Most interventions were delivered within the United States of America (*n* = 43, 74.1%). The most common study design reported was Randomised Control Trial (*n* = 27, 46.6%). Included studies involved synchronous telehealth interventions targeting nutrition (11, 18.9%), physical activity (5, 8.6%) or nutrition and physical activity (41, 70.7%) and were delivered predominately via videoconference (*n* = 17, 29.3%).

**Conclusions:**

Despite differences in intervention characteristics, the number of randomised control trials published suggests sufficient scope for future systematic reviews to determine intervention effectiveness related to nutrition and physical activity telehealth interventions for rural populations.

**Trial registration:**

The scoping review protocol was not pre-registered.

**Supplementary Information:**

The online version contains supplementary material available at 10.1186/s12966-023-01505-2.

## Background

Chronic diseases including cardiovascular disease, cancer, chronic respiratory disease, and diabetes are among the leading causes of morbidity and mortality worldwide [1]. Smoking, poor nutrition, at-risk alcohol consumption, and physical inactivity (SNAP) are key behavioural risk factors in the development of these chronic diseases and their reduction is listed as the third objective on the World Health Assembly’s *WHO Global action plan for the prevention and control of Noncommunicable Diseases (NCDs) 2013–2020* [1–3]. Health behaviour change interventions are organized sets of activities designed to change specific health behaviours, such as SNAP behaviours [4]. Effective behaviour change interventions are often complex, and can target varying populations, communities, or individuals [4–10]. They can also incorporate different behaviour change techniques and methods of delivery depending on the intervention context [4–10]. This can make them challenging to replicate or implement across different contexts, such as between urban and rural areas [4].

People living in rural areas, defined broadly as any area outside of a major city, can experience unique challenges accessing health services and professionals that provide individualised behaviour change interventions as part of their routine care [10, 11]. Geographical distance impacts rural health worker recruitment and retention, resulting in a shortage of healthcare professionals in rural areas worldwide [12–15]. This shortage can result in increased travel distances for rural people accessing health services, higher cost of private services and longer wait times for public services [11, 15]. Challenges accessing healthcare services, in combination with lower socio-economic factors, contribute to the higher prevalence of risky lifestyle behaviours and overall poorer health outcomes experienced by rural populations compared to their metropolitan counterparts [11]. It is therefore important to understand how service models such as telehealth, which may be used to connect rural populations with healthcare providers, can be utilised to improve health behaviours, and overall health outcomes, in these populations.

Telehealth is defined as ‘delivery of health services, where distance is a critical factor, by health professionals using information and communication technologies (ICT)’ [16]. The term telehealth is often used interchangeably with telemedicine [17]. However telemedicine refers to the delivery of medical, diagnostic and treatment related services, usually by doctors, whilst telehealth includes a wider variety of remote healthcare services, including behaviour change interventions often delivered by allied health workers [17]. Telehealth has been consistently shown to be effective for healthcare delivery and has additional benefits for rural communities [18–21]. These benefits include improved access to and increased quality of clinical care, reduced overall cost of service delivery, reduced demand for emergency services, reduced travel time for both rural patients and health professionals, improved management of chronic and complex conditions, and improved professional development opportunities for rural staff which may contribute to improved rural medical workforce recruitment and retention [22–25].

Despite these benefits, uptake of telehealth into mainstream rural health service delivery has been slow [23]. Proposed barriers to implementation include initial cost of set up, [25] inconsistent government rebates for telehealth, [26] lack of education and training for clinicians, [24, 27, 28] limited knowledge of the changes in provider-patient interactions as a result of altered communication patterns, [29] lack of clinician skill with technology, and concerns with insurance and liability [19]. Additionally, clinicians, clients and service providers may view telehealth as a lesser service compared to face-to-face models [19].

Research into the implementation of telehealth into rural health services has largely focused on medical interventions in acute care settings [23]. A 2016 systematic review summarising currently published telehealth studies in rural Australia found nearly 60% (*n* = 41) of studies were for medical interventions alone and only 13% (*n* = 9) investigated intervention delivery by allied health professionals [23]. The type of allied health professionals delivering the interventions was not specified in the review nor was there any description of any behaviour change interventions provided [23]. To our knowledge there is no recent review scoping the literature on telehealth delivery of SNAP lifestyle interventions in rural areas.

The aim of this current scoping review is to begin to address this evidence gap, by summarising the characteristics of studies investigating synchronous telehealth interventions targeting two SNAP lifestyle behaviours of nutrition and physical activity for adults living in rural areas. The purpose of this is to contribute to understanding of the scope of studies in this area and provide a foundation for systematic review of intervention effectiveness in the future. In this review, nutrition and physical activity interventions have been separated from smoking and alcohol interventions due to acknowledgment that lifestyle behaviour change techniques may vary if the targeted behaviour involves an addictive substance (alcohol or smoking) or not (physical activity and nutrition) [4–10].

### Objectives

To summarise the characteristics of studies investigating synchronous telehealth interventions targeting nutrition and physical activity behaviours in adults living in rural areas. This will include addressing the following questions:

1. What is the scope of research describing nutrition and physical activity interventions delivered using telehealth to rural populations, including number of citations retrieved and their included interventions, reported publication date and study design?
2. What are the characteristics of included interventions, including intervention settings, methods and reported outcomes?
3. What theories are referenced in current literature, including implementation or behaviour change theories?
4. Does the current body of literature contain enough homogenous study design to warrant systematic review?

## Methods

### Protocol and registration

The methods of this scoping review were guided by the PRISMA extension for scoping review checklist (Additional file 1) [30]. The scoping review protocol was not pre-registered.

### Eligibility criteria

Studies eligible for inclusion during title/abstract screening included adult participants aged 18 years and over living in rural areas and involved a synchronous (video or phone consultation) telehealth intervention that addressed smoking and/or alcohol and/or physical activity and/or nutrition. Studies involving mHealth, defined by the World Health Organisation (WHO) as ‘medical and public health practice supported by mobile devices, such as mobile phones, patient monitoring devices, Personal Digital Assistants (PDAs), and other wireless devices’, and face-to-face interventions were included if they also had a synchronous phone or video telehealth component [31].

A comparator was not necessary for inclusion and all study designs except for systematic and scoping reviews were eligible. Studies were limited to high income countries as classified by the World Bank and all outcomes were considered [32].

‘Rural’ was defined by the included study authors and may have included but was not limited to descriptions such as ‘rural’, ‘regional’, ‘remote’ or ‘non-metropolitan’. This was to account for the lack of international definition of ‘rural’.

Studies were excluded if they were duplicates; were not in English; were not set in a high-income country; did not include a lifestyle intervention or a synchronous telehealth component; were not set in a rural area or present data from rural populations separately; were systematic/scoping reviews; included populations currently undergoing acute care treatment; included paediatric populations or were published outside of the included date range. This inclusion/exclusion criteria was re-applied to studies retrieved for full-text screening. Studies addressing smoking and/or alcohol were excluded after full text screening.

### Information sources

Electronic databases searched included MEDLINE, EMBASE, COCHRANE, PSYCHINFO, SCOPUS, CINAHL, Web of Science and INFORMIT. A search of the reference lists of included articles and retrieved systematic reviews was also completed. All databases were searched up to June 2023.

### Search strategy

The search strategy was developed by the research team and in consultation with the medical librarian. The search consisted of Medical Subject Headings (MeSH) and focussed keywords. MeSH terms included telemedicine, remote consultation, health behaviour, diet, diet therapy, nutrition therapy, eating, exercise, exercise therapy, physical therapy modalities, sedentary behaviour, alcohol drinking, tobacco smoking, smoking cessation, smoking reduction, smoking, obesity, obesity management, weight loss, diabetes mellitus type 2, cardiovascular diseases, chronic disease, rural health services, rural health, rural population, and regional health planning. The term telehealth was included under the telemedicine MeSH. The full MEDLINE search strategy can be found in Additional file 2.

### Data management

All records were exported from databases into an endnote library and deduplicated. Remaining records were uploaded to Covidence (Veritas Health Innovation, Melbourne, Australia. Available at www.covidence.org) for additional de-duplication, abstract and full text screening.

### Selection process

Two independent reviewers completed abstract and full text screening of all articles. Conflicts were discussed and resolved between independent reviewers or resolved by a third reviewer.

### Data collection process

Data extraction was completed by one reviewer and checked by another. Variables collected for each study included study title, author, year of publication, study design, National Health and Medical Research Council (NHMRC) Evidence Hierarchy categorisation of study design, aim, setting (country of intervention delivery), rural definition provided, participant inclusion/exclusion criteria, lifestyle risk factor targeted, methods of intervention delivery, qualification of intervention facilitator, timing of intervention including number of telehealth consults, length of consults, and duration of the intervention, theory referenced, outcomes measured and whether there were statistically significant findings reported [33].

### Data synthesis

Data relating to nutrition and physical activity behaviours will be summarised using descriptive statistics. Percentages will be calculated from the number of included studies when describing study design and intervention characteristics, and from the number of included publications when describing reported outcomes and theoretical frameworks referenced.

## Results

### Citation retrieval

**Fig. 1:**
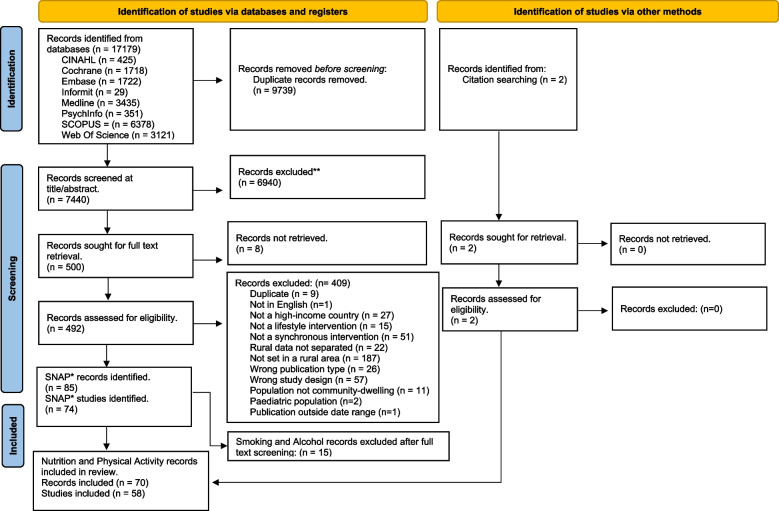
Map of the identification and screening of studies for review inclusion [32]. *SNAP: Smoking, Nutrition, Alcohol and Physical Activity. From: Page MJ, McKenzie JE, Bossuyt PM, Boutron I, Hoffmann TC, Mulrow CD, et al. The PRISMA 2020 statement: an updated guideline for reporting systematic reviews. BMJ 2021;372:n71. https://doi.org/10.1136/bmj.n71. For more information, visit: http://www.prisma-statement.org/

The search strategy identified 17179 citations across eight databases (Fig. 1). After duplication and title/abstract screening 500 citations were eligible for full text retrieval. Of these citations a total of 85 publications (*n* = 73 studies) were identified as eligible with 70 publications (*n* = 58 studies) identified for non-addictive behaviours (nutrition and physical activity) and 15 publications (*n* = 13 studies) for addictive behaviours (smoking and alcohol). The 15 publications (*n* = 13 studies) identified for smoking and alcohol were excluded at this stage and the characteristics of the 70 publications (*n* = 58 studies) for nutrition and physical activity are described here.

### Publication dates

Included nutrition and/or physical activity records were published from 1999–2023, with 63 (90%) records published from 2010 onward and approximately a third (*n* = 22, 32.4%) records published since the COVID-19 pandemic from 2020 onward (see Additional files 3 and 4) [34–94].

### Study design

Of the 58 included studies, Randomised Control Trial (RCT) was the most common study design (*n* = 27, 46.6%), [35, 37–39, 43–45, 52, 54–56, 58, 59, 61, 65, 69–73, 79–81, 95–97] followed by pre-post (*n* = 11, 19.0%) [50, 60, 63, 64, 66, 77, 78, 86, 98–101]. Study design of remaining studies included cohort (retrospective and prospective) (*n* = 7, 12.1%), [34, 36, 47, 48, 83–85, 88] single arm trial (*n* = 6, 10.3%), [40, 41, 46, 67, 82, 89, 92, 102, 103] case study (*n* = 3, 5.2%), [57, 104, 105] pseudo-RCT (*n* = 2, 3.4%), [42, 51] non-inferiority trial (*n* = 1, 1.7%) and qualitative (*n* = 1, 1.7%) [49, 76]. Study designs were categorised according to the NHMRC Evidence Hierarchy as level II (*n* = 27, 46.6%), level III study (*n* = 28, 50.9%), level IV (*n* = 14, 24.2%) or not at all (*n* = 1, 1.7%) see Additional files 3 and 5) [35, 37–39, 43–45, 49, 52, 54–56, 58, 59, 61, 65, 69–73, 79–81, 95–97].

### Intervention characteristics

Intervention characteristics are summarised in Table 1, with additional information included in Additional file 3.

**Table 1:** Summary of intervention characteristics of included studies

*Intervention characteristics*	*Number of studies (n, (%))*	*Publications (n, (%))*
***Lifestyle risk factor targeted***		
*Physical activity*	5 (8.6)	6 (8.6)
*Nutrition*	11 (18.9)	12 (17.1)
*Physical activity and nutrition*	41 (70.7)	51 (72.9)
*Unspecified*	1 (1.7)	1 (1.4)
***Method of delivery***		
*Phone*	12 (20.6)	13 (18.6)
*Videoconference*	17 (29.3)	21 (30.0)
*Phone/videoconference*	8 (13.8)	9 (12.9)
*Phone/videoconference/face-to-face*	2 (3.4)	2 (2.9)
*Phone/videoconference/mHealth*	2 (3.4)	2 (2.9)
*Phone/face-to-face*	5 (8.6)	6 (8.6)
*Phone/face-to-face/mHealth*	1 (1.7)	1 (1.4)
*Videoconference/face-to-face*	9 (15.5)	12 (17.1)
*Videoconference/face-to-face/mHealth*	1 (1.7)	1 (1.4)
*Unspecified*	1 (1,7)	3 (4.3)
***Mode of delivery***		
*Group*	16 (27.6)	17 (24.3)
*Individual*	24 (41.4)	32 (45.7)
*Group and individual*	17 (29.3)	20 (28.6)
*Unspecified*	1 (1.7)	1 (1.4)
***Delivered by a health professional?***		
*Yes*	47	57
*Dietitian*	*26*	*30*
*Nurse Practitioner*	*16*	*21*
*Exercise professional*	*11*	*12*
*Psychologist*	*9*	*10*
*Health/Lifestyle Coach*	*5*	*7*
*Diabetes Educator*	*5*	*5*
*Physiotherapist*	*4*	*5*
*Community health worker*	*3*	*3*
*Social Worker*	*3*	*3*
*Counsellor/Health Educator*	*2*	*2*
*Individuals with tertiary training in a Health Science*	*2*	*2*
*Endocrinologist*	*1*	*1*
*Nutrition student supervised by dietitian*	*1*	*1*
*Unspecified Allied Health Clinician*	*1*	*2*
*Occupational Therapist*	*1*	*1*
*Pharmacist*	*1*	*1*
*Not specified*	*11*	*13*
***Total number of telehealth consults delivered during intervention***		
< *10*	20 (34.5)	24 (34.3)
*10–20*	15 (25.9)	18 (25.7)
*20* +	8 (13.8)	10 (14.3)
*Patient preference*	3 (5.2)	5 (7.1)
*Not specified*	13 (22.4)	13 (18.6)
***Range of consult length (mins)***		
*Not reported*	25 (43.1)	29 (41.4)
*15–60*	26 (44.8)	33 (47.1)
> *60*	7 (12.1)	8 (11.4)
***Reported outcome(s)***		
*Behaviour change (diet/physical activity)*	40	48
*Morbidity*	11	13
*Anthropometry/weight loss*	36	40
*Biochemistry*	22	26
*Feasibility*	17	22
*Qualitative themes*	7	7
*Cost-effectiveness*	2	2

### Settings

The majority of included studies were set in rural areas of the United States of America (USA) (*n* = 43, 74.1%), [34, 36, 37, 40–48, 51–55, 57–59, 62–66, 69–71, 73, 77–82, 86–92, 95–97, 100, 104, 105] with the remaining set in rural areas of Canada (*n* = 6, 11.5%), [35, 50, 60, 67, 68, 75, 76, 99] Australia (*n* = 6, 10.3%), [38, 39, 72, 74, 83–85, 93, 94] United Kingdom (UK) (*n* = 2, 3.4%), [49, 56] and Taiwan (*n* = 1, 1.7%) (see Additional files 3 and 6). [61] A definition of rurality was reported in 19 (32.8%) studies. [42–44, 46, 50, 52, 57–60, 67, 70, 75, 77, 78, 81, 85, 88, 92, 96, 97, 103] Nine studies (15.5%) used an official measure of rurality that was recognised by the country in which the study was conducted, and 10 studies (17.2%) reported an unofficial measure (see Additional files 6 and 7) [42–44, 46, 50, 52, 57, 58, 60, 67, 68, 70, 74, 75, 77, 78, 81, 85, 88, 92, 96, 97].

### Intervention methods

Most study interventions (*n* = 41, 70.7%) targeted nutrition and physical activity simultaneously [34–37, 40–44, 46, 48–52, 54–57, 60, 62, 63, 65–67, 70–74, 76–82, 84–95, 99, 103]. Videoconference was the most popular method of telehealth intervention delivery. (*n* = 38, 65.5%) [34, 35, 40, 41, 43, 46–54, 57–64, 66, 67, 69, 71, 73, 75–80, 82, 86, 88, 91, 95, 99, 100, 103–105]. In some studies videoconference was used in conjunction with phone (*n* = 8, 13.7%), [35, 43, 49, 69, 73, 80, 104] face-to-face (9, 15.5%), [40, 41, 47, 50, 51, 61, 62, 66, 71, 82, 86, 95] phone plus face-to-face (*n* = 2, 3.3%), [52, 88] or a combination of phone and/or face to face and/or mHealth methods (*n* = 3, 5.2%) [84, 87, 89]. Telehealth delivery was reported as an adjunct to in-person delivery in *n* = 17 (29.3%) included studies [40, 41, 44, 47, 50–52, 61, 70, 71, 82, 84–86, 88, 93–96, 102, 106].

Most interventions included one-on-one delivery to individuals as the only intervention mode (*n* = 24, 41.4%), [36–39, 45, 47, 55–59, 64, 67, 72–74, 79–81, 84, 85, 87, 89, 91–94, 97, 100, 103, 104] or in combination with a group component (*n* = 17, 29.3%) [35, 40–42, 44, 48, 52, 62, 65, 66, 70, 71, 75, 95, 96, 105]. Most interventions (*n* = 49, 80.3%) were delivered by one or more health professionals or health/lifestyle coaches, [34–52, 55, 57–59, 61, 62, 64, 65, 67, 69, 70, 82, 84, 85, 87–95, 103–105] with the most utilised being dietitians (*n* = 26, 44.8%), [34, 35, 40–49, 52, 55, 57–59, 61, 64, 65, 71, 77–79, 82, 85, 88, 95, 104, 105] nurses (*n* = 16, 27.6%), [35–37, 47, 48, 52, 57–59, 61, 79, 80, 84, 85, 87, 88, 92–94, 96] and exercise professionals (either scientist or physiologists) (*n* = 11, 19%) [35, 44, 46–48, 51, 65, 71, 77, 85, 95].

The total number of telehealth consults delivered throughout included interventions ranged from a single session (*n* = 3, 4.9%), [63, 100, 106] to up to 78 sessions (*n* = 1, 1.7%) [40, 41]. The intervention that delivered 78 telehealth sessions included 26 weekly nutrition consultations and 52 bi-weekly telehealth exercise consultations delivered over a 26-week period [40, 41]. Twenty interventions delivered less than 10 consultations over the reported intervention period whilst 23 interventions delivered 10 consultations or more [36–39, 46, 50, 51, 56, 59–61, 63, 66, 72, 80, 82, 85–92, 94, 96, 100]. The frequency of delivery of 13 studies was not described, whilst three interventions were delivered at varied frequencies depending on patient preference [45, 47, 57, 59, 64, 69, 77–79, 83, 84, 93, 97, 101, 104, 105, 107]. Timing of telehealth consultations ranged from 15 min to 2 h with nutrition consultations generally shorter (15–60 min) and physical activity consultations generally longer (60–120 min).

### Reported Outcomes

Many publications reported multiple primary outcomes (see Table 1). Primary outcomes related to participant behaviour change (*n* = 48, 68.6%); [36, 38–42, 45, 47–57, 59, 60, 62–69, 71, 72, 75, 77–81, 87–93, 95–97, 99] disease status (*n* = 13, 18.6%); [47, 51, 59, 61, 63, 73, 79, 87, 93, 96, 97] anthropometric markers (weight loss) (*n* = 40, 57.1%); [34, 36, 37, 40–46, 48, 51, 52, 54, 55, 58, 59, 61–65, 82, 87, 95] biochemistry (*n* = 26, 44.8%); [36, 44–47, 51, 52, 55, 58, 59, 61–64, 69, 71, 73, 74, 77–79, 88, 90, 93–96] intervention feasibility (*n* = 22, 31.4%); [35, 40, 41, 47, 50, 57, 64, 66–68, 83–90, 92, 99, 100] participant experience measured through qualitative methods (*n* = 7, 10%), [35, 49, 50, 53, 62, 76, 92], and cost-effectiveness (*n* = 2, 2.9%) [38, 43].

### Reference to a theorical framework

Twenty-five publications (32.1%) referenced a theory, theoretical framework, or theoretical model in the description of the intervention methods (see Table 2) [37–43, 45, 49, 52, 55, 56, 66–69, 81, 83, 84, 86, 87, 89, 91, 92, 95]. Nine of these (12.5%) referenced more than one theory [40, 41, 45, 52, 55, 66, 81, 86, 89]. A total of ten theories were referenced with the most common theories relating to behaviour change. These included the social cognitive theory (*n* = 9), [37, 40–43, 55, 66, 86, 89] self-determination theory (*n* = 6), [37–43, 49, 55, 66–68, 86, 89] and trans-theoretical model of behaviour change (*n* = 5) [45, 52, 56, 69, 91].

**Table 2:** Summary of theories referenced in included studies

*Theory*	*Number of studies*	*References*
*Social Cognitive Theory* [108]	9	[37, 40–43, 55, 66, 86, 89]
*Self-determination theory* [109]	6	[38, 39, 49, 67, 68, 92]
*Technology Acceptance model* [110]	2	[40, 41]
*Health belief model* [111]	2	[45, 52]
*Transtheoretical model of behaviour change* [112]	5	[6, 45, 52, 56, 91]
*The Reach, Effectiveness, Adoption, Implementation, Maintenance (RE-AIM) framework* [113]	2	[55, 81]
*Self-regulation theory* [114]	1	[95]
*The Obesity-Related Behavioural Intervention Trials (ORBIT) model* [115]	2	[66, 86]
*Theory of planned behaviour* [116]	1	[81]
*Model for large scale knowledge translation*	2	[83, 84]

## Discussion

The aim of the current scoping review is to summarise the characteristics of studies investigating synchronous telehealth interventions targeting non-addictive, nutrition and physical activity behaviours in adults living in rural areas. The purpose of this was to increase understanding of the extent, range, and nature of studies in this area, and provide a foundation for systematic review of these studies in the future. Publications included in the current review were identified as a part of a larger database search which also included addictive behaviour interventions targeting smoking and alcohol behaviours in rural populations. However, studies reporting addictive behaviours were separated at data analysis to be reported independently, due to consideration of the varying behaviour change techniques that may be more effective in behaviours involving addictive substances [4–10]. Overall, this current scoping review included a substantial number of RCT’s (*n* = 27, 46.6%), highlighting that the current scope of research in this area warrants future systematic reviews to determine intervention effectiveness.

Of the many publications (*n* = 70, 58 studies) identified for inclusion in this review, the majority were published after 2010. The current review identified a proliferation of publications between 2020 and 2022. This increase coincides with the COVID-19 pandemic and demonstrates a shift toward telehealth research to meet the demands of physical distancing during the pandemic years [16]. There appears to be a drop in publications from 2022–2023, however, this may reflect the current review only including studies published before June 2023 (Additional file 4).

Only five of the 80 high-income countries were represented in the current review with the USA, Canada and Australia accounting for the highest proportion of published studies [32]. These countries are among the top 10 highest income countries in the world, and have large geographical distances between metropolitan and rural/remote areas. In particular, Canada and Australia both have very small populations per land mass (four and three people per square kilometre of land respectively) [32, 117]. As such, impetus, and availability of resources for adopting evidence-based telehealth services into healthcare delivery may drive a higher output of studies in these areas. However, this small number of represented countries highlights a limitation in the current scope of research and should be considered in future interpretations of study effectiveness and transferability across rural settings in different countries.

This review identified that a wide range of definitions of rurality were used by the included studies, which may result in varying population characteristics and rural settings [118]. In some studies, rurality was stated, but not defined, making it difficult to ascertain population characteristics or study settings. In studies that did provide a definition of rurality, only nine used an official definition of rurality recognised by the country of intervention setting. Within these nine studies, six varying official definitions of rurality are described [42–44, 46, 58, 59, 70, 74, 81, 97]. Such differences in definitions have been shown to result in a wide range of intervention contexts, including varying population characteristics such as education and poverty status, population density and access to health services [118]. The use of official measures of rurality in the design of future rural telehealth interventions will make identification of included population characteristics easier to understand and assist in transferability of intervention designs across contexts.

Included studies demonstrated key similarities across intervention characteristics. A large proportion of the included studies targeted nutrition and physical activity simultaneously. This is not surprising, given weight loss was a commonly reported outcomes of included studies, and improving dietary intake and physical activity are nationally recommended strategies for weight management [119–122].

Key differences identified across intervention characteristics included consultation lengths (ranging from 15 min to over 60 min), consultation frequencies (ranging from a single consultation up to 78 consultations) and combinations of delivery methods (telehealth alone or in combination with face-to-face or mHealth methods). There are currently no guidelines outlining optimum intervention intensity for nutrition and physical activity behaviour change interventions, nor is it clear whether telehealth interventions are more effective as a standalone or complimentary service to more traditional face-to-face models. A systematic review of reported outcomes stratified by intervention characteristics may identify which characteristics are more effective for nutrition and physical activity behaviour change.

While future systematic reviews are warranted, a potential limitation to consider is the inconsistent reporting of behaviour change outcomes across included studies. Despite all included studies describing the delivery of behaviour change interventions, only 40 studies (48 publications) reported behaviour change variables such as changes in diet or physical activity levels as a study outcome. This means the effectiveness of the behaviour change interventions on nutrition and physical activity levels in the remaining 18 studies (25 publications) cannot be assessed.

Approximately one third of included publications (*n* = 25) referenced a theoretical framework and most of these publications (*n *= 23) referenced theory related to behaviour change. Only two studies referenced an implementation theory, framework, or model, suggesting a lack of consideration of implementation science in current literature. This is a limitation of included studies, as implementation factors such as poor internet connectivity, cost of set-up and lack of healthcare professional telehealth training are cited as key barriers to the uptake of telehealth universally, not just in rural areas [24]. Use of implementation science can inform study design, ensuring implementation factors are considered across a diverse range of contexts [123]. This may be particularly relevant in a rural setting, where context can vary greatly, and implementation strategies need to be adaptable in order to sustain telehealth services [23].

### Strengths

To our knowledge there are no other reviews summarising the scope of research investigating nutrition and physical activity interventions delivered using telehealth to adults living in rural areas. A previous systematic review published in 2016 synthesised literature on telehealth services in rural Australia and aimed to synthesise factors associated with successful and sustainable telehealth services [23]. Whilst it included a large number of articles (*n* = 116), only nine related to allied health and there was no distinction as to whether these services provided nutrition or physical activity interventions [23]. A more recent systematic review has described the effectiveness of telehealth individual video-conference interventions in reducing smoking, nutrition, alcohol, physical activity, and obesity risk factors in an adult population, and included RCTs from both metropolitan and rural areas [108]. However, of the included studies, only four were set in rural locations, and only two were related to physical activity and none were related to nutrition [108]. The current scoping review is the first to summarise current literature on this topic and lays a foundation for future systematic reviews on this topic in the future.

### Limitations

A scoping review study design can result in the identification of a large volume of literature; however, the quality of evidence is not appraised. Furthermore, a scoping review design does not synthesise outcomes to assess intervention effectiveness. The search strategy of this scoping review was limited to studies in the English language and only included interventions that were published. It is likely that many other synchronous telehealth physical activity and nutrition services exist in practise, but have not been published [23]. Lastly, there were many studies that included a synchronous telehealth nutrition and/or physical activity intervention in areas that may have included a rural population, however, the results of these studies were not stratified by rurality. Studies that did not stratify by rurality were not included as it was impossible to ascertain whether they included the target population.

## Conclusion

The current scoping review summarised literature on telehealth nutrition and/or physical activity interventions delivered to adults living in rural areas of high-income countries. A large volume of literature was identified and included a predicted spike in intervention numbers coinciding with the COVID-19 pandemic. Included studies were largely from rural areas of the USA, with definitions of rurality varying across studies. This may result in variation across what is defined as a rural context, and comparison between study outcomes in the future difficult. Whilst study designs varied, the current review identified 27 published RCTs for physical activity and nutrition, providing a strong foundation for future systematic review of study quality, outcomes, and overall intervention effectiveness. Reported outcomes stratified by intervention characteristics (e.g., length, frequency, and mode of delivery) may identify which telehealth delivery method is most effective for rural populations. Lastly, there is limited reference to implementation or behaviour change theories in the included intervention designs. This indicates an area of potential improvement in future intervention study design of telehealth interventions targeting improved nutrition and physical activity levels in rural populations.

### Supplementary Information

**Additional file 1.** Preferred Reporting Items for Systematic reviews and Meta-Analysis extension for Scoping Reviews (PRISMA-ScR) Checklist.PRISMA checklist outlining page numbers of completed sections in manuscript. 

**Additional file 2.** Medline search strategy and initial results for current scoping review.Medline search strategy and initial results for current scoping review presented in table format.

**Additional file 3.** Characteristics of included studies. Summary table describing the extracted characteristics of each included study.

**Additional file 4.** Number of studies and year of publication since 1999. Visual representation of number of studies published each year since 1999.

**Additional file 5.** Classification of included studies into National Health and Medical Research Council (NHMRC) categories.

**Additional file 6.** Measure of rurality provided per country of intervention setting. Bar graph describing the measure of rurality provided per country, and distinguishing whether the measure was official or unofficial.

**Additional file 7.** Official measure of rurality featured in included studies. Description of the official measures of rurality referenced in included studies.

## Data Availability

The datasets used and/or analysed during the current study are available from the corresponding author on reasonable request.
