# Supplementary material for: Delivery of telehealth nutrition and physical activity interventions to adults living in rural areas: a scoping review
Source: Int J Behav Nutr Phys Act. 2023 Sep 15;20:110. doi: 10.1186/s12966-023-01505-2 (PMC10504780; doi:10.1186/s12966-023-01505-2)
Supplement: Supplementary file 2 — Additional file 2. Medline search strategy and initial results for current scoping review.Medline search strategy and initial results for current scoping review presented in table format. [file 12966_2023_1505_MOESM2_ESM.docx]

Supplementary table 2 Medline search strategy

| \| [**# ▲**](https://ovidsp-dc2-ovid-com.ezproxy.newcastle.edu.au/ovid-b/ovidweb.cgi?&S=BHCAFPLPPLEBPDDCJPPJDGHGABCIAA00&Sort+Sets=descending) \| **Searches** \| \| --- \| --- \| \| 1 \| telehealth.mp. or Telemedicine/ \| \| 2 \| Remote Consultation/ or videoconsult*.mp. \| \| 3 \| phone consult*.mp. \| \| 4 \| telecare.mp. \| \| 5 \| mhealth.mp. \| \| 6 \| ehealth.mp. \| \| 7 \| 1 or 2 or 3 or 4 or 5 or 6 \| \| 8 \| lifestyle intervention.mp. \| \| 9 \| exp Health Behavior/ or health behaviour.mp. \| \| 10 \| well-being.mp. \| \| 11 \| wellness.mp. \| \| 12 \| diet.mp. or exp Diet/ or exp Diet Therapy/ \| \| 13 \| exp Nutrition Therapy/ or nutrition.mp. \| \| 14 \| food intake.mp. or Eating/ \| \| 15 \| exp Exercise/ or exercise.mp. or exp Exercise Therapy/ \| \| 16 \| physical activit*.mp. \| \| 17 \| physical therap*.mp. or Physical Therapy Modalities/ \| \| 18 \| Sedentary Behavior/ or sedentary behavio?r.mp. \| \| 19 \| non-sedentary behavio?r.mp. \| \| 20 \| Alcohol intake.mp. or Alcohol Drinking/ \| \| 21 \| exp Tobacco Smoking/ or exp Smoking Cessation/ or smoking.mp. or exp Smoking Reduction/ or exp Smoking/ \| \| 22 \| obesity management.mp. or exp Obesity/ or exp Obesity Management/ \| \| 23 \| weight loss.mp. or Weight Loss/ \| \| 24 \| Diabetes Mellitus, Type 2/ or diabetes.mp. \| \| 25 \| cardiovascular disease.mp. or Cardiovascular Diseases/ \| \| 26 \| chronic disease.mp. or Chronic Disease/ \| \| 27 \| 8 or 9 or 10 or 11 or 12 or 13 or 14 or 15 or 16 or 17 or 18 or 19 or 20 or 21 or 22 or 23 or 24 or 25 or 26 \| \| 28 \| exp Rural Health Services/ or rural.mp. or exp Rural Health/ or exp Rural Population/ \| \| 29 \| rural commun*.mp. \| \| 30 \| regional health planning.mp. or exp Regional Health Planning/ \| \| 31 \| regional health.mp. \| \| 32 \| regional population.mp. \| \| 33 \| regional commun*.mp. \| \| 35 \| remote population.mp. \| \| 36 \| remote commun*.mp. \| \| 37 \| 28 or 29 or 30 or 31 or 32 or 33 or 34 or 35 or 36 \| \| 38 \| 7 and 27 and 37 \| \| 39 \| limit 38 to english language \| |
| --- | --- | --- | --- | --- | --- | --- | --- | --- | --- | --- | --- | --- | --- | --- | --- | --- | --- | --- | --- | --- | --- | --- | --- | --- | --- | --- | --- | --- | --- | --- | --- | --- | --- | --- | --- | --- | --- | --- | --- | --- | --- | --- | --- | --- | --- | --- | --- | --- | --- | --- | --- | --- | --- | --- | --- | --- | --- | --- | --- | --- | --- | --- | --- | --- | --- | --- | --- | --- | --- | --- | --- | --- | --- | --- | --- | --- | --- | --- |
