# Supplementary material for: Delivery of telehealth nutrition and physical activity interventions to adults living in rural areas: a scoping review
Source: Int J Behav Nutr Phys Act. 2023 Sep 15;20:110. doi: 10.1186/s12966-023-01505-2 (PMC10504780; doi:10.1186/s12966-023-01505-2)
Supplement: Supplementary file 3 — Additional file 3. Characteristics of included studies. Summary table describing the extracted characteristics of each included study. [file 12966_2023_1505_MOESM3_ESM.docx]

Supplementary table 3 Characteristics of studies included in scoping review of smoking, nutrition, alcohol and physical activity telehealth interventions

| **Study name** | **Authors** | **Year** | **Country** | **Study design** | **Qualified facilitator** | **Lifestyle Risk Factors** | | **Method of delivery** | | | | | | **Primary outcome** | | | | | | |
| --- | --- | --- | --- | --- | --- | --- | --- | --- | --- | --- | --- | --- | --- | --- | --- | --- | --- | --- | --- | --- |
|  |  |  |  |  |  | **Nutrition** | **Physical Activity** | **Phone** | **Video** | **In person** | **mHealth** | **Group** | **Individual** | **Behaviour change** | **Morbidity** | **Weight** | **Biochemistry** | **Feasibility** | **Qualitative** | **Cost-**  **effectiveness** |
| ¡Vivir Mi Vida! (Live My Life!, or ¡VMV!) | Niemiec et al. (101) | 2021 | USA | Single arm non-RCT | X | X | X |  | X | X |  | X | X | X |  | X | X |  | X |  |
| CHAP project | Beleigoli et al.(82) | 2021 | Aus | Other |  |  |  |  |  |  |  |  |  |  |  |  |  | X |  |  |
|  | Beleigoli et al.(83) | 2022 | Aus | Prospective Cohort | X | X | X |  | X | X | X |  | X |  |  |  |  | X |  |  |
| Cookin' Up Health | Tessaro et al. (105) | 2007 | USA | RCT |  | X |  | X |  |  |  |  | X | X | X |  |  |  |  |  |
| CyberX | Elder et al. (97) | 2016 | USA | Pre-post |  |  | X |  | X |  |  | X |  | X |  |  |  |  | X |  |
| Diabetes Telecare | Davis et al. (51) | 2010 | USA | RCT | X | X | X | X | X | X |  | X | X | X |  | X | X |  |  |  |
| ENHANCeD | Benson et al. (106) | 2019 | USA | RCT | X | X |  | X |  |  |  |  | X | X |  | X | X |  |  |  |
| HEALTHH | Prochaska et al. (107) | 2018 | USA | RCT | X | X |  | X | X |  |  | X |  | X |  | X | X |  |  |  |
|  | Oppezzo et al. | 2022 | USA | RCT | X | X | X | X | X |  |  |  | X | X |  |  |  |  |  |  |
| Healthy Lifestyle Program | Batsis et al. (108) | 2019 | USA | Single-armed Non-RCT | X | X | X |  | X | X |  | X | X | X |  | X |  | X |  |  |
|  | Batsis et al. (109) | 2021 | USA | Single-armed Non-RCT | X | X | X |  | X | X |  | X | X | X |  | X |  | X |  |  |
| Healthy4U | Barrett et al. (37) | 2019 | Aus | RCT | X |  | X | X |  |  |  |  | X | X |  |  |  |  |  | X |
|  | Barrett et al. (38) | 2018 | Aus | RCT | X |  | X | X |  |  |  |  | X | X |  |  |  |  |  |  |
| HeLP-her | Wang et al. (110) | 2018 | Aus | RCT |  | X | X | X |  | X | X | X | X |  | X | X |  |  |  |  |
| IDEATel | Homenko et al. (111) | 2010 | USA | RCT | X | X |  |  | X |  |  |  | X |  |  | X | X |  |  |  |
|  | Izquierdo et al. (112) | 2010 | USA | RCT | X | X |  |  | X |  |  |  | X | X | X | X | X |  |  |  |
|  | West et al. (113) | 2010 | USA | RCT | X | X | X |  | X |  |  |  | X | X | X |  | X |  |  |  |
| MODERN | Yiallourou et al.(92) | 2021 | Aus | RCT | X | X | X | X |  | X |  |  | X | X | X | X | X |  |  |  |
|  | Carrington and Zimmet (93) | 2017 | Aus | RCT | X | X | X | X |  | X |  |  | X |  |  | X | X |  |  |  |
| MOST-TR | Taylor et al. (114) | 2009 | Can | Pre-post |  | X | X |  | X |  |  | X |  | X |  |  |  | X |  |  |
|  | Taylor et al. (115) | 2012 | Can | Qualitative | | X | X |  | X |  |  | X |  |  |  |  |  |  | X |  |
| MOVE! | Ahrendt et al. (116) | 2014 | USA | Retrospective Cohort | X | X | X |  | X |  |  | X |  |  |  | X |  |  |  |  |
| Unnamed | Banner et al. (34) | 2015 | Can | RCT | X | X | X | X | X |  |  | X | X |  |  |  |  | X | X |  |
| Unnamed | Barker et al. (117) | 2016 | USA | Prospective Cohort | X | X | X | X |  |  |  |  | X | X |  | X | X |  |  |  |
| Unnamed | Barnason et al. (118) | 2019 | USA | RCT | X | X | X | X |  |  |  |  | X |  |  | X |  |  |  |  |
| Unnamed | Befort et al. (119) | 2010 | USA | PseudoRCT | X | X | X | X |  |  |  | X | X | X |  | X |  |  |  |  |
| Unnamed | Brown et al. (120) | 2020 | USA | Single arm non-RCT | X | X | X |  | X |  |  | X |  |  |  | X | X |  |  |  |
| Unnamed | Ciemins et al. (121) | 2018 | USA | Prospective Cohort | X | X | X |  | X |  |  | X | X | X |  | X |  |  |  |  |
| Unnamed | Ciemins et al. (46) | 2011 | USA | Prospective Cohort | X | X |  |  | X | X |  |  | X | X | X |  | X | X |  |  |
| Unnamed | Cliffe et al. (48) | 2021 | UK | Qualitative | X | X | X | X | X |  |  | X |  | X |  |  |  |  | X |  |
| Unnamed | Dal Bello-Haas et al. (49) | 2014 | Can | Pre-post | X |  | X |  | X | X |  | X |  | X |  |  |  | X | X |  |
| Unnamed | Dalleck et al. (50) | 2011 | USA | Non-RCT | X | X | X |  | X | X |  | X |  | X | X | X | X |  |  |  |
| Unnamed | Fazzino et al. (53) | 2017 | USA | RCT |  | X | X |  | X |  |  | X |  | X |  | X |  |  |  |  |
| Unnamed | Gillham et al. (55) | 2010 | UK | RCT |  | X | X | X |  |  |  |  | X | X |  |  |  |  |  |  |
| Unnamed | Johnson et al. (103) | 2001 | USA | Case-study | X | X |  | X | X |  |  |  | X |  |  |  |  |  |  |  |
| Unnamed | Liou et al. (60) | 2013 | Tai | RCT | X | X |  |  | X | X |  | X |  |  | X | X | X |  |  |  |
| Unnamed | McCabe et al. (104) | 2001 | USA | Case study | X | X |  |  | X |  |  | X | X |  |  |  |  |  |  |  |
| Unnamed | Nuovo, J (62) | 2013 | USA | Pre-post |  | X | X |  | X |  |  | X |  | X | X | X | X |  |  |  |
| Unnamed | Olfert et al. (63) | 2019 | USA | Pre-post | X | X |  |  | X |  |  |  | X | X |  | X | X | X |  |  |
| Unnamed | Perri et al.(64) | 2020 | USA | RCT | X | X | X | X |  |  |  | X | X | X |  | X |  |  |  |  |
| Unnamed | Radcliff et al. (69) | 2012 | USA | RCT | X | X | X | X |  | X |  | X | X |  |  | X |  |  |  |  |
| Unnamed | Sangster et al. (71) | 2015 | Aus | RCT |  | X | X | X |  |  |  |  | X | X |  | X |  |  |  |  |
| Unnamed | Skelly et al. (95) | 2009 | USA | RCT | X | X |  | X |  | X |  | X | X | X | X | X | X |  |  |  |
| Unnamed | Stiles et al. (99) | 1999 | USA | Pre-post | X | X |  |  | X |  |  |  | X |  |  |  |  | X |  |  |
| Unnamed | Vadheim et al. (76) | 2010 | USA | Pre-Post | X | X | X |  | X |  |  | X |  | X |  | X | X |  |  |  |
| Unnamed | Vadheim et al. (77) | 2017 | USA | Pre-post | X | X | X |  | X |  |  | X |  | X |  | X | X |  |  |  |
| Unnamed | Young et al. (79) | 2014 | USA | RCT | X | X | X | X | X |  |  |  | X | X |  |  |  |  |  |  |
| Unnamed | Batsis et al. (81) | 2022 | USA | Single arm non-RCT | X | X | X |  | X | X |  | X | X |  |  | X |  |  |  |  |
| Unnamed | Champion et al. (84) | 2022 | Aus | Prospective Cohort | X | X | X | X |  | X |  |  | X |  |  |  |  | X |  |  |
| Unnamed | Johansson et al. (86) | 2023 | USA | RCT | X | X | X | X | X |  | X |  | X | X | X | X | X | X |  |  |
| Unnamed | Ladner et al. (87) | 2022 | USA | Prospective Cohort | X | X | X | X | X | X |  | X |  | X |  | X | X | X |  |  |
| Unnamed | Li et al. (88) | 2022 | USA | Single arm non-RCT | X | X | X | X | X |  | X |  | X | X |  |  |  | X |  |  |
| PA and FV in rural-living Yas | Price et al. (67) | 2020 | Can | Single arm non-RCT | X | X | X |  | X |  |  |  | X | X |  |  |  | X |  |  |
|  | Price and Brunet (66) | 2021 | Can | Single arm non-RCT | X | X | X |  | X |  |  |  | X | X |  |  |  | X |  |  |
|  | Price and Brunet (91) | 2022 | Can | Single arm non-RCT | X | X | X |  |  |  |  |  | X | X |  |  |  | X | X |  |
| PRISM | Holloway et al. (56) | 2011 | USA | Case-study | X | X | X |  | X |  |  |  | X | X |  |  |  | X |  |  |
| REPOWER | Befort et al. (43) | 2021 | USA | RCT | X | X | X | X |  | X |  | X | X |  |  | X | X |  |  |  |
| Rural LEAP | O'Neal et al. (89) | 2022 | USA | RCT | X | X | X | X |  |  |  | X | X | X |  | X |  | X |  |  |
| Rural women connecting for better health | Befort et al. (42) | 2016 | USA | RCT | X | X | X | X | X |  |  | X |  |  |  | X |  |  |  | X |
| RVTRI | Levy et al. (100) | 2015 | USA | Retrospective Pre-Post | X |  | X | X | X |  |  |  | X | X |  |  |  | X |  |  |
| SipsmartER | Zoellner et al. (80) | 2018 | USA | RCT |  | X | X | X |  |  |  |  | X | X |  | X |  |  |  |  |
| SOLAR | Actrn, (122) | 2021 | USA | RCT | X | X | X | NS | NS | NS | NS |  | X |  | X |  |  |  |  |  |
| Tele-CDSMP | Jaglal et al. (59) | 2013 | Can | Pre-post |  | X | X |  | X |  |  | X |  | X |  |  |  |  |  |  |
| Telehealth-PR | Stickland et al. (74) | 2011 | Can | Non-inferiority trial |  |  | X |  | X |  |  | X | X | X |  |  |  |  |  |  |
| The Shape Program | Foley et al. (54) | 2012 | USA | RCT | X | X | X | X |  |  |  |  | X | X |  | X | X |  |  |  |
| TOURs | Perri et al. (94) | 2008 | USA | RCT | X | X | X |  | X | X |  | X | X | X |  | X | X |  |  |  |
|  | Rickel et al. (70) | 2011 | USA | RCT | X | X | X |  | X | X |  | X | X | X |  | X | X |  |  |  |
| TREAT | Siminerio et al. (123) | 2015 | USA | RCT | X | X | X | X | X |  |  |  | X |  | X | X | X |  |  |  |
| weSurvive | Porter et al. (65) | 2021 | USA | Pre-post |  | X | X |  | X | X |  | X | X | X |  | X |  | X |  |  |
|  | Dennett et al. (85) | 2023 | USA | Pre-post |  | X | X |  | X | X |  | X | X |  |  |  |  | X |  |  |
