# Supplementary material for: Delivery of telehealth nutrition and physical activity interventions to adults living in rural areas: a scoping review
Source: Int J Behav Nutr Phys Act. 2023 Sep 15;20:110. doi: 10.1186/s12966-023-01505-2 (PMC10504780; doi:10.1186/s12966-023-01505-2)
Supplement: Supplementary file 4 — Additional file 4. Number of studies and year of publication since 1999. Visual representation of number of studies published each year since 1999. [file 12966_2023_1505_MOESM4_ESM.docx]

Supplementary figure 1 Number of studies and year of publication since 1999
