# Supplementary material for: Delivery of telehealth nutrition and physical activity interventions to adults living in rural areas: a scoping review
Source: Int J Behav Nutr Phys Act. 2023 Sep 15;20:110. doi: 10.1186/s12966-023-01505-2 (PMC10504780; doi:10.1186/s12966-023-01505-2)
Supplement: Supplementary file 5 — Additional file 5. Classification of included studies into National Health and Medical Research Council (NHMRC) categories. [file 12966_2023_1505_MOESM5_ESM.docx]

Supplementary table 4 Number of included studies in each NHMRC evidence hierarchy study design category

| ***Evidence level*** | ***Study designs included*** | ***Number of studies (n (%))*** | ***Number of publications (n (%))*** |
| --- | --- | --- | --- |
| *II* | RCT | 27 (46.6) | 33 (47.1) |
| *III* | Cohort | 7 (12.1) | 7 (10) |
|  | Single arm trial | 6 (10.3) | 9 (12.9) |
|  | PseudoRCT | 2 (3.4) | 2 (2.9) |
|  | Non-inferiority trial | 1 (1.7) | 1 (1.4) |
|  | Pre-post | 11 (19) | 12 (17.1) |
| *IV* | Case-study | 3 (5.2) | 3 (4.3) |
|  | Qualitative | 1 (1.7) | 2 (2.9) |
| *NA* | Intervention development | NA | 1 (1.4) |
