# Supplementary material for: Delivery of telehealth nutrition and physical activity interventions to adults living in rural areas: a scoping review
Source: Int J Behav Nutr Phys Act. 2023 Sep 15;20:110. doi: 10.1186/s12966-023-01505-2 (PMC10504780; doi:10.1186/s12966-023-01505-2)
Supplement: Supplementary file 6 — Additional file 6. Measure of rurality provided per country of intervention setting. Bar graph describing the measure of rurality provided per country, and distinguishing whether the measure was official or unofficial. [file 12966_2023_1505_MOESM6_ESM.docx]

Supplementary figure 2 Definition of rurality provided per country of intervention setting
