# Supplementary material for: Delivery of telehealth nutrition and physical activity interventions to adults living in rural areas: a scoping review
Source: Int J Behav Nutr Phys Act. 2023 Sep 15;20:110. doi: 10.1186/s12966-023-01505-2 (PMC10504780; doi:10.1186/s12966-023-01505-2)
Supplement: Supplementary file 7 — Additional file 7. Official measure of rurality featured in included studies. Description of the official measures of rurality referenced in included studies. [file 12966_2023_1505_MOESM7_ESM.docx]

Supplementary table 5 Official definitions of rurality described in included studies

| Official measures of rurality | Definition of rurality | Study (n) | Reference |
| --- | --- | --- | --- |
| **United States Census Bureau definition (1)** | All population, housing and territory not included in an Urban Area (UA)/Urban Cluster (UC) (<10000 people) | 1 | (2) |
| **Rural Urban Commuting Area Codes (RUCA) (3)** | 10 categories spanning urban metropolitan to rural non-metro. Incorporates population and commuting data as a proxy for ‘connectedness’. Values greater than or equal to 4 were considered rural. | 2 | (4), (5) |
| **Rural-Urban Continuum Codes (RUCC) (6)** | 9 categories spanning urban metropolitan to rural non-metro. Values greater than or equal to 7 were considered rural for all tracts within the county. | 1 | (7) |
| **Health Resource Services Administration definition (HRSA) (8)** | All tracts with RUCAs greater than or equal to 4 | 1 | (9) |
| **Medically Underserved Areas (MUAs) (10)** | Geographic areas and populations with a lack of access to primary care services. | 4 | (11),(12),(13, 14) |
| **Health Professional Shortage Areas (HPSA) (10)** | Geographic areas, populations, or facilities with a shortage of primary, dental, or mental health care providers. | 3 | (12),(13),(15) |
| **Access/Remoteness Index of Australia (ARIA) (16, 17)** | Categorises remoteness in Australia based on access to services. Categories include Very Remote Australia, Remote Australia, Outer Regional Australia, Inner Regional Australia and Major Cities of Australia | 1 | (18) |

1. Bureau USC. Rural America: How does the U.S. Census Bureau Define "Rural"? United States of America: U.S Census Bureau; 2023 [Available from: <https://mtgis-portal.geo.census.gov/arcgis/apps/MapSeries/index.html?appid=49cd4bc9c8eb444ab51218c1d5001ef6#:~:text=The%20Census%20Bureau%20defines%20rural,rural%20based%20on%20this%20definition>.

2. Befort CA, Donnelly JE, Sullivan DK, Ellerbeck EF, Perri MG. Group versus individual phone-based obesity treatment for rural women. Eating Behaviors. 2010;11(1):11-7.

3. Service USDoAER. Rural-Urban Commuting Area Codes United States: U.S. Department of Agriculture; 2023 [Available from: <https://www.ers.usda.gov/data-products/rural-urban-commuting-area-codes/>.

4. Befort CA, Klemp JR, Sullivan DK, Shireman T, Diaz FJ, Schmitz K, et al. Weight loss maintenance strategies among rural breast cancer survivors: The rural women connecting for better health trial. Obesity (Silver Spring). 2016;24(10):2070-7.

5. Befort CA, Vanwormer JJ, Desouza C, Ellerbeck EF, Gajewski B, Kimminau KS, et al. Effect of Behavioral Therapy with In-Clinic or Telephone Group Visits vs In-Clinic Individual Visits on Weight Loss among Patients with Obesity in Rural Clinical Practice: A Randomized Clinical Trial. JAMA - Journal of the American Medical Association. 2021;325(4):363-72.

6. Service USDoAER. Rural-Urban Continuum Codes United States: U.S. Department of Agriculture; 2023 [Available from: <https://www.ers.usda.gov/data-products/rural-urban-continuum-codes.aspx>.

7. Zoellner JM, You W, Estabrooks PA, Chen Y, Davy BM, Porter KJ, et al. Supporting maintenance of sugar-sweetened beverage reduction using automated versus live telephone support: findings from a randomized control trial. International journal of behavioral nutrition and physical activity. 2018;15(1):N.PAG.

8. Administration HRaS. Defining Rural Population United States of America2022 [Available from: <https://www.hrsa.gov/rural-health/about-us/what-is-rural#:~:text=The%20Census%20does%20not%20define,UCs)%20of%202%2C500%20%2D%2049%2C999%20people>.

9. Brown JD, Hales S, Evans TE, Turner T, Sword DO, O'Neil PM, et al. Description, utilisation and results from a telehealth primary care weight management intervention for adults with obesity in South Carolina. J Telemed Telecare. 2020;26(1-2):28-35.

10. Administration HRS. What is a Shortage Designation? [Available from: <https://bhw.hrsa.gov/workforce-shortage-areas/shortage-designation#mups>.

11. Izquierdo R, Lagua CT, Meyer S, Ploutz-Snyder RJ, Palmas W, Eimicke JP, et al. Telemedicine intervention effects on waist circumference and body mass index in the IDEATel project. Diabetes Technol Ther. 2010;12(3):213-20.

12. Homenko DR, Morin PC, Eimicke JP, Teresi JA, Weinstock RS. Food insecurity and food choices in rural older adults with diabetes receiving nutrition education via telemedicine. J Nutr Educ Behav. 2010;42(6):404-9.

13. Tessaro I, Rye S, Parker L, Mangone C, McCrone S. Effectiveness of a Nutrition Intervention with Rural Low-income Women. American Journal of Health Behavior. 2007;31(1):35-43.

14. Ladner KA, Berry SR, Hardy J. Increasing Access to Diabetes Education in Rural Alabama Through Telehealth. Am. 2022;122(9):39-47.

15. Radcliff TA, Bobroff LB, Lutes LD, Durning PE, Daniels MJ, Limacher MC, et al. Comparing Costs of Telephone vs Face-to-Face Extended-Care Programs for the Management of Obesity in Rural Settings. J Acad Nutr Diet. 2012;112(9):1363-73.

16. Statistics ABo. The Australian Statistical Geography Standard (ASGS) Remoteness structure 2021 [Available from: <https://www.abs.gov.au/websitedbs/d3310114.nsf/home/remoteness+structure>.

17. Statistics ABo. Remoteness Structure: Australian Statistical Geography Standard (ASGS) Edition 3 Australia2023 [Available from: <https://www.abs.gov.au/statistics/standards/australian-statistical-geography-standard-asgs-edition-3/jul2021-jun2026/remoteness-structure>.

18. Ski CF, Vale MJ, Bennett GR, Chalmers VL, McFarlane K, Jelinek VM, et al. Improving access and equity in reducing cardiovascular risk: the Queensland Health model. Medical Journal of Australia. 2015;202(3):148-52.
